# Gastric rupture caused by intragastric perforation of splenic artery aneurysm: a case report and literature review

**DOI:** 10.1186/s40792-024-01944-4

**Published:** 2024-06-17

**Authors:** Hazuki Koguchi, Keita Nakatsutsumi, Takahiro Ikuta, Akihiro Fujita, Yasuhiro Otomo, Koji Morishita

**Affiliations:** 1 Department of Emergency and Disaster Medicine, Trauma and Acute Critical Care Center, Tokyo Medical and Dental University, 1-5-45, Yushima, Bunkyo-Ku, Tokyo, 113-0034 Japan; 2 Department of Critical Care Medicine and Trauma, National Hospital Organization Disaster Medical Center, Tokyo, Japan

**Keywords:** Acute care surgery, Damage control surgery, Gastric cancer, Gastric rupture, Ruptured splenic artery aneurysm

## Abstract

**Background:**

The rupture of splenic artery pseudoaneurysm (SAP) is life-threatening disease, often caused by trauma and pancreatitis. SAPs often rupture into the abdominal cavity and rarely into the stomach.

**Case presentation:**

A 70-year-old male with no previous medical history was transported to our emergency center with transient loss of consciousness and tarry stools. After admission, the patient become hemodynamically unstable and his upper abdomen became markedly distended. Contrast-enhanced computed tomography performed on admission showed the presence of a splenic artery aneurysm (SAP) at the bottom of a gastric ulcer. Based on the clinical picture and evidence on explorative tests, we established a preliminary diagnosis of ruptured SAP bleeding into the stomach and performed emergency laparotomy. Intraoperative findings revealed the presence of a large intra-abdominal hematoma that had ruptured into the stomach. When we performed gastrotomy at the anterior wall of the stomach from the ruptured area, we found pulsatile bleeding from the exposed SAP; therefore, the SAP was ligated from inside of the stomach, with gauze packing into the ulcer. We temporarily closed the stomach wall and performed open abdomen management, as a damage control surgery (DCS) approach. On the third day of admission, total gastrectomy and splenectomy were performed, and reconstruction surgery was performed the next day. Histopathological studies of the stomach samples indicated the presence of moderately differentiated tubular adenocarcinoma. Since no malignant cells were found at the rupture site, we concluded that the gastric rupture was caused by increased internal pressure due to the intra-abdominal hematoma.

**Conclusions:**

We successfully treated a patient with intragastric rupture of the SAP that was caused by gastric cancer invasion, accompanied by gastric rupture, by performing DCS. When treating gastric bleeding, such rare causes must be considered and appropriate diagnostic and therapeutic strategies should be designed according to the cause of bleeding.

## Background

Rupture of splenic artery pseudoaneurysm (SAP) is a fatal condition [1–3]. SAPs are often caused by trauma and pancreatitis and bleed into the abdominal cavity, rarely rupturing into the stomach [4–8]. Herein, we report a rare case of SAP caused by gastric cancer invading the splenic artery, which ruptured into the stomach with hemorrhagic shock, resulting in gastric rupture because of increased intragastric pressure due to hematoma.

## Case presentation

**Fig. 1:**
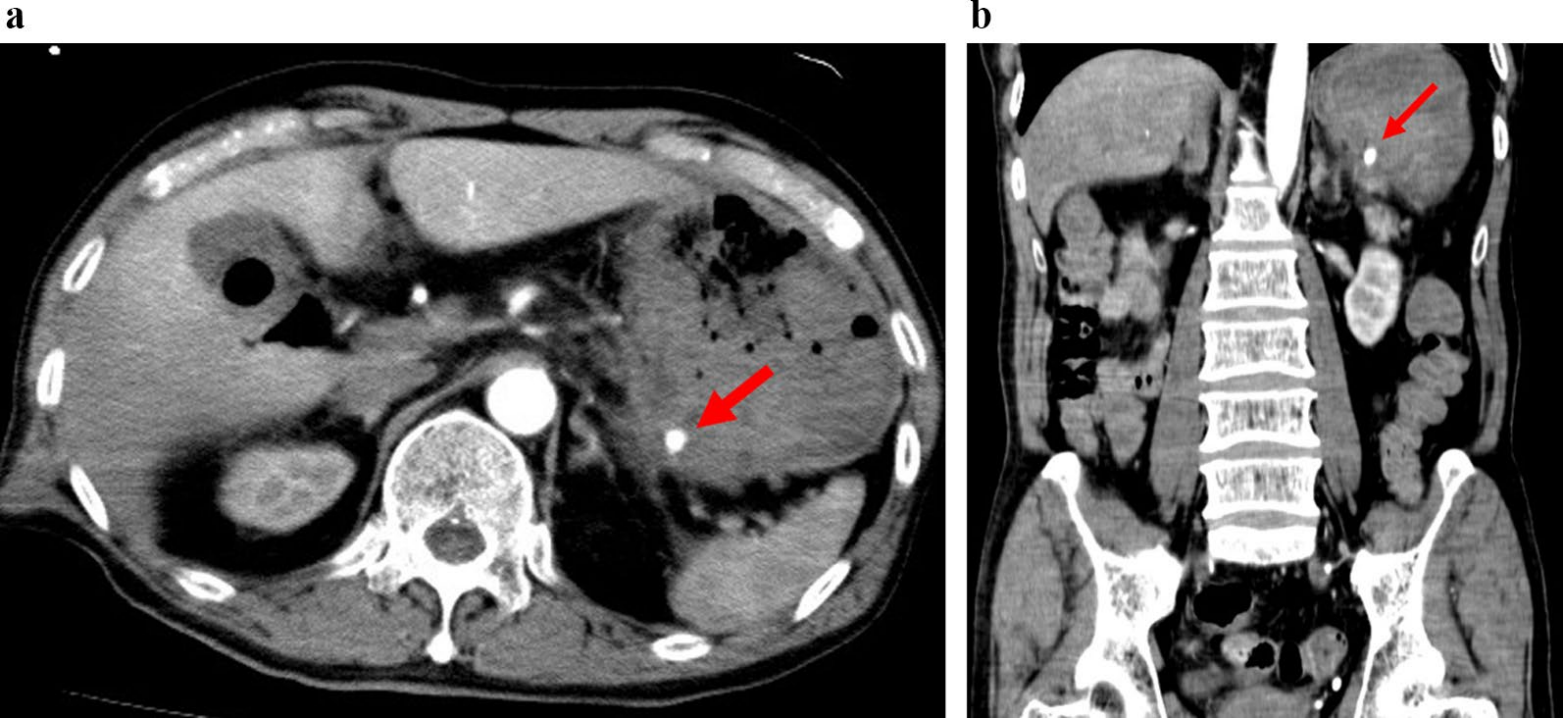
**a** Axial computed tomography showing a splenic artery aneurysm (red arrow) that ruptured into the stomach on admission. **b** Coronal computed tomography showing a splenic artery aneurysm (red arrow) that ruptured into the stomach on admission

**Fig. 2:**
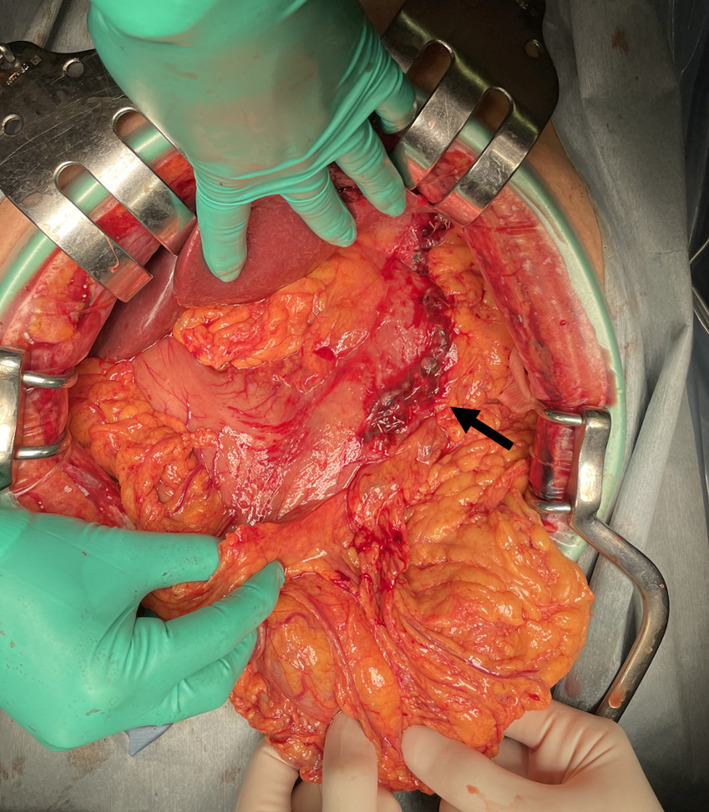
Ruptured gastric wall temporally closed in the second surgery (black arrow)

**Fig. 3:**
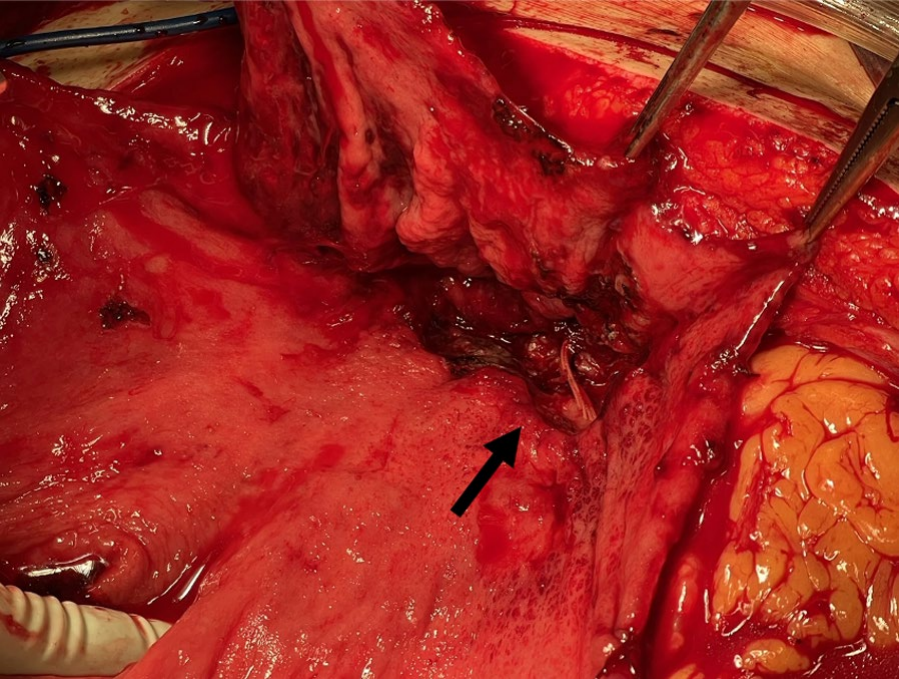
Ligated splenic artery aneurysm at the bottom of the gastric ulcer in the second surgery (black arrow)

A 70-year-old male with no previous medical history was transported by ambulance to our emergency center with transient loss of consciousness and tarry stools. This patient was a smoker, not obese, and had no history of habitual eating of smoked or salted foods. The patient had mildly clouded consciousness, with pale skin and cold sweats; his upper abdomen was distended, and he had tarry stools at the emergency department on arrival. The patient’s vital signs were as follows: heart rate (HR), 138/min; blood pressure (BP), 92/59 mmHg; SpO2, 99% for oxygen setting of 6 L/min; respiratory rate, 17/min; body temperature, 36.5℃. We suspected hemorrhagic shock due to upper gastrointestinal bleeding (UGIB), given the severe anemia with serum hemoglobin of 4.6 mg/dL and tarry stools. Therefore, we performed endotracheal intubation and emergency upper gastrointestinal endoscopy with blood transfusion. However, we could not observe distal to the esophagogastric junction owing to the presence of a filling hematoma. After the transfusion of six units of red blood cell, the patient’s vital signs stabilized (BP, 120/60 mmHg; HR, 90/min). Then, contrast-enhanced computed tomography (CT) was performed, followed by admission to the intensive care unit; we planned to repeat the endoscopy at a later date. Six hours after the admission, the patient become hemodynamically unstable again, and his upper abdomen became markedly distended. The contrast-enhanced CT performed on admission showed an SAP at the bottom of a gastric ulcer; therefore, the diagnosis of UGIB due to rupture of the SAP was confirmed (Fig. 1a, b). Despite massive transfusion, the vital signs remained unstable. Therefore, we selected emergency laparotomy rather than interventional radiology or upper gastrointestinal endoscopy as a hemostatic procedure. Resuscitative endovascular balloon occlusion of the aorta (REBOA) was placed in Zone 1, and the aorta was completely occluded until the operation room was prepared. At the start of surgery, since the patient was experiencing life-threatening bleeding (BP, 50/35 mmHg; HR, 145/min), with arterial blood pH of 7.10, we decided to perform damage control surgery (DCS). Intraoperative findings revealed a large intra-abdominal hematoma, resulting in a gastric rupture of approximately 10 cm in the anterior wall of the middle gastric body. When we performed gastrotomy from the anterior wall of the stomach from the ruptured area to the cardia, we found pulsatile bleeding from the SAP exposed at the bottom of the ulcer on the posterior wall of the upper gastric body. We ligated the artery aneurysm from inside of the stomach and performed gauze packing into the ulcer. The stomach wall was temporarily closed and open abdomen management was performed (Figs. 2, 3). The total perioperative transfusion volume was 36 units of red blood cells, 38 units of fresh frozen plasma, and 40 units of platelets.

On the third day of admission, second-look laparotomy was performed. Intraoperative findings during the second-look laparotomy indicated that the rupture in the gastric wall was too wide to be closed directly or via partial gastrectomy. In addition, the area of the splenic artery invaded by the gastric cancer was close to the splenic hilum. Therefore, we decided to perform a total gastrectomy with splenectomy. Roux-en-Y reconstruction was performed during the third-look laparotomy the next day. On the 8th day of admission, the patient showed clear consciousness and was extubated. After treatment of an intra-abdominal abscess, probably due to minor leakage of esophageal jejunal anastomosis, with antibiotics and drainage of the abscess, the patient was transferred to the hospital for rehabilitation on the 52nd day of admission.

Histopathological examination of the stomach samples indicated the diagnosis of the moderately differentiated tubular adenocarcinoma. Since no malignant cells were found at the rupture site and no other specific reason of gastric rupture in the gastric wall had been proven pathologically, we concluded that the increased intragastric pressure due to the hematoma had led to gastric rupture.

## Discussion

The main causes of SAPs are trauma and pancreatitis [4]. Herein, the SAP formed at the bottom of the ulcer in a patient with gastric cancer had ruptured, which is rarely caused by direct invasion of cancer or ulcer [5–7]. SAPs often rupture intraperitoneally, perforating the gastrointestinal tract in less than 30% of the cases, and perforation of the stomach is even rarer, occurring in approximately 10% of the cases [8]. Bleeding of ruptured SAPs into free spaces such as the digestive tract or abdominal cavity can be fatal [9–12]. The guidelines of the Society for Vascular Surgery recommend treating SAPs with transarterial embolization (TAE) or surgery, depending on hemodynamic, location of the aneurysm, and facility capabilities [13]. In the present case, emergency surgery was performed because of the patient's unstable condition. However, if the SAP was diagnosed immediately after the CT scan, before the patient had become hemodynamically unstable, TAE could have been an effective option for hemostasis and emergency surgery could have been avoided.

Gastric rupture has been reported to be caused by trauma injuries [14–16]; binge-eating [17–22]; intake of excessive sodium bicarbonate [23, 24] in psychiatric patients, obstruction of the intestinal tract (e.g., superior mesenteric artery syndrome) [25–29]; and iatrogenic causes such as bag-mask ventilation, cardiopulmonary resuscitation, and esophageal intubation [30–36]. The most likely location of rupture is the lesser curvature, followed by the anterior wall. The lesser curvature is susceptible to rupture because of the weak gastric wall with few mucosal folds and poor mobility afforded by the hepatogastric ligament [23, 32]. Gastric dilation is commonly caused by the atony of gastric wall, acute gastric dilation, and mechanical obstruction such as superior mesenteric artery syndrome, resulting in gastric rupture by ischemic necrosis of the gastric wall [22, 25, 26, 37].

In our patient’s case, pathological examination revealed no invasion of cancer at the site of the rupture, and CT imaging on admission did not show gastric rupture. Therefore, the patient was diagnosed with excessive increase in intragastric pressure because of gastric rupture caused by a hematoma. The gastric rupture led to the loss of the tamponade effect, temporarily controlling the bleeding, which resulted in re-bleeding and sudden collapse of the patient’s vital signs after admission.

DCS is widely used for resuscitation of critically ill patients with severe traumatic injuries. Since patients with coagulopathy, acidosis, and/or hypothermia are at high risk of death, these conditions are included under the term “deadly triad”. DCS is a series of procedures wherein bleeding, contamination, and abdominal hypertension are controlled first; initial surgery is completed as soon as possible; and definitive surgery is performed after the management of the “deadly triad” and stabilization of the patient’s general condition in the intensive care unit [38–43]. Recently, DCS has also been reported to be useful in non-traumatic abdominal emergencies, such as intra-abdominal hemorrhage, peritonitis with septic shock, hollow viscus perforation, acute mesenteric ischemia, necrotizing enterocolitis, acute pancreatitis, and abdominal compartment syndrome [44–51]. We selected DCS, because our patient was in imminent cardiac arrest with metabolic acidosis at the time of the start of surgery and definitive surgery was expected to be complicated and lengthy. Following the principle of DCS, we performed the initial surgery only to control hemorrhage, stabilized the patient’s general condition in the intensive care unit, and then surgically reconstructed the stomach. Thus, the patient’s life was saved after this three-stage operation.

The efficacy of REBOA has also been reported in cases of hemorrhagic shock from subdiaphragmatic organs, for both traumatic and non-traumatic hemorrhage, as a method for controlling the inflow of bleeding before emergency hemostatic surgery [12, 52–54]. However, the indications for REBOA are controversial, as REBOA could delay the time to hemostasis, resulting in poor outcome [55]. In our patient’s case, REBOA was inserted until the operation room was prepared and did not delay the time to hemostasis. Therefore, REBOA helped prevent cardiac arrest due to hemorrhage and secure the field of view during the operation.

## Conclusion

We successfully treated a patient with intragastric rupture of the SAP that was caused by gastric cancer invasion, accompanied by gastric rupture, using DCS. When treating gastric bleeding, rare causes, such as perforation of SAP, must be considered, and appropriate diagnostic and therapeutic strategies must be determined according to the cause of bleeding.

## Data Availability

The data that support the findings of this study are available on request from the corresponding author.

## Abbreviations

SAP: Splenic artery pseudoaneurysm
HR: Heart rate
BP: Blood pressure
UGIB: Upper gastrointestinal bleeding
CT: Computed tomography
REBOA: Resuscitative endovascular balloon occlusion of the aorta
DCS: Damage control surgery

## Availability of data and materials

The data that support the findings of this study are available on request from the corresponding author.
